# Ultra-high on-chip optical gain in erbium-based hybrid slot waveguides

**DOI:** 10.1038/s41467-019-08369-w

**Published:** 2019-01-25

**Authors:** John Rönn, Weiwei Zhang, Anton Autere, Xavier Leroux, Lasse Pakarinen, Carlos Alonso-Ramos, Antti Säynätjoki, Harri Lipsanen, Laurent Vivien, Eric Cassan, Zhipei Sun

**Affiliations:** 1 Department of Electronics and Nanoengineering, Aalto University, Tietotie 3, FI-00076 Espoo, Finland; 2 Centre for Nanoscience and Nanotechnology (C2N), CNRS, Université Paris-Sud, Université Paris-Saclay, UMR 9001, 91405 Orsay Cedex, France; 3 Optoelectronics Research Centre, University of Southampton, University Road, Southampton, Hampshire, SO17 1BJ UK; 4 Institute of Photonics, University of Eastern Finland, FI-80101 Joensuu, Finland; 5 QTF Centre of Excellence, Department of Applied Physics, Aalto University, FI-00076 Espoo, Finland

## Abstract

Efficient and reliable on-chip optical amplifiers and light sources would enable versatile integration of various active functionalities on the silicon platform. Although lasing on silicon has been demonstrated with semiconductors by using methods such as wafer bonding or molecular beam epitaxy, cost-effective mass production methods for CMOS-compatible active devices are still lacking. Here, we report ultra-high on-chip optical gain in erbium-based hybrid slot waveguides with a monolithic, CMOS-compatible and scalable atomic-layer deposition process. The unique layer-by-layer nature of atomic-layer deposition enables atomic scale engineering of the gain layer properties and straightforward integration with silicon integrated waveguides. We demonstrate up to 20.1 ± 7.31 dB/cm and at least 52.4 ± 13.8 dB/cm net modal and material gain per unit length, respectively, the highest performance achieved from erbium-based planar waveguides integrated on silicon. Our results show significant advances towards efficient on-chip amplification, opening a route to large-scale integration of various active functionalities on silicon.

## Abstract (web summary)

Realizing efficient on-chip amplification in silicon is challenging due to either non-compatible integration or small gain per unit length of the amplifier material. Here, the authors report ultra-high on-chip optical gain in erbium-based hybrid silicon nitride slot waveguides with a monolithic, CMOS-compatible and scalable atomic-layer deposition process.

## Introduction

Over the past decades, silicon integrated photonics has emerged as one of the most studied and developed fields in modern photonics and optoelectronics^1^. The ultimate reason for this is to improve the well-developed microelectronic technologies by integrating optical functionalities on the integrated circuit chips. One way to realize such improvement is to replace the metallic interconnects in the microchips with optical interconnects to provide faster response time, wider transmission bandwidth, and lower power consumption^2^. In fact, major electronics manufacturers, such as IBM and Intel, have already started to use silicon integrated photonics in their high-bitrate systems to overcome the signal integrity issues introduced by the time delay within the copper interconnects^3^. Therefore, silicon integrated photonics is believed to be the key technology in some of the most important communication systems, such as data routers, where transfer rates can exceed well above Tbit/s^4^.

In silicon integrated photonics, most of the passive monolithic building blocks, including couplers, splitters, and resonators have already been demonstrated. However, some of the essential active on-chip functionalities, such as light emission and amplification are still challenging to be realized due to the indirect band gap nature of silicon. Solutions relying on hybrid integration of III/V lasers on silicon, although developed in the past years^5^, bring additional complexity to the fabrication processes and do not provide the long-awaited monolithic approach necessary for the massive development of optical interconnects and on-chip optical signal processing. This has set drawbacks for the development of next-generation hybrid integrated circuits, where electrical and optical functions are anticipated to be combined together for the best possible cost-effective and high-performance technology.

On the other hand, one of the most straightforward approaches to enable on-chip optical signal generation and amplification at telecom wavelengths is to rely on solutions previously developed by the optical fiber community, i.e., exploiting rare-earth (e.g., erbium)-doped waveguide amplifiers. Indeed, erbium-doped integrated waveguide amplifiers and lasers have shown excellent potential for such purposes, especially at the important C-band (1530–1565 nm) wavelength regime due to their long excited state lifetime and integratability with silicon^6^. For example, an on-chip internal net gain of ~20 dB over a 12.9 cm long Er:Al_2_O_3_ ridge waveguide amplifier has been demonstrated for a −38 dBm input signal power^7^. Furthermore, silicon-compatible Er:Al_2_O_3_ microring and distributed feedback lasers have also been reported^8–10^. Despite their superior performance, the average gain per unit length of the typical erbium-based integrated devices yet remains relatively small (up to few dB/cm) as the erbium-concentration is limited to less than one atomic percent due to quenching and up-conversion effects of active ions at higher concentration levels^11–15^. Consequently, the small gain per unit length makes it very challenging to realize efficient on-chip optical amplification in silicon integrated waveguides where the propagation losses are typically on the order of dB/cm. Additionally, the demonstrated large footprint of cm long amplifiers is hardly compatible with the need for compact silicon photonic circuit integration.

Here, we report ultra-high on-chip optical gain by integrating atomic scale engineered erbium-doped aluminum oxide directly on silicon nitride slot waveguides with a monolithic, CMOS-compatible and scalable atomic-layer deposition (ALD) process. We observe up to ~20.1 ± 7.31 dB/cm net modal gain per unit length and at least ~52.4 ± 13.8 dB/cm net material gain per unit length in sub-mm long hybrid Er:Al_2_O_3_–Si_3_N_4_ slot waveguides. With the proposed configuration, we show that it is possible to convert passive silicon integrated waveguides directly into efficient active on-chip amplifiers with a single and straightforward process, enabling novel and simple integration of various active functionalities on the silicon platform.

## Results

### Waveguide fabrication

In this work, we fabricated and studied Er:Al_2_O_3_–Si_3_N_4_ hybrid slot waveguides. Silicon nitride (Si_3_N_4_) was chosen as the passive waveguide material due to its several advantages compared to silicon, including higher transparency at wavelengths below 1.1 μm, ultra-low two-photon absorption effect at telecom wavelengths and ultimately, smaller propagation losses. These properties have made silicon nitride a widely used platform in silicon integrated photonic applications^16,17^. The fabrication process of the device chip consisted of two steps: (1) fabrication of the passive silicon nitride waveguides and (2) deposition of the active Er:Al_2_O_3_-layer on the as-fabricated waveguides. The passive silicon nitride waveguides were defined on an 8″ silicon wafer with a 500 nm thick  thermal oxide. A 5.5 μm thick layer of optical quality silicon dioxide, followed by a 460 nm thick layer of silicon nitride was deposited on the substrate. The silicon dioxide was deposited with low-pressure chemical vapor deposition, whereas the silicon nitride was deposited with plasma-enhanced chemical vapor deposition. To proceed with the process, a 500 nm thick ZEP-520 resist was spin-coated onto the substrate, baked and then exposed with electron beam lithography (80 kV, 2.1 nA). The exposed resist was then removed to dry-etch the silicon nitride for the designed waveguide pattern. The dry-etching was done with inductively coupled plasma (SF_6_/C_4_F_8_). Finally, the resist was removed to reach the final design of the waveguide structure. After the passive waveguide fabrication, the wafer was cut into smaller yet identical chips and the processing proceeded for a single chip.

**Fig. 1:**
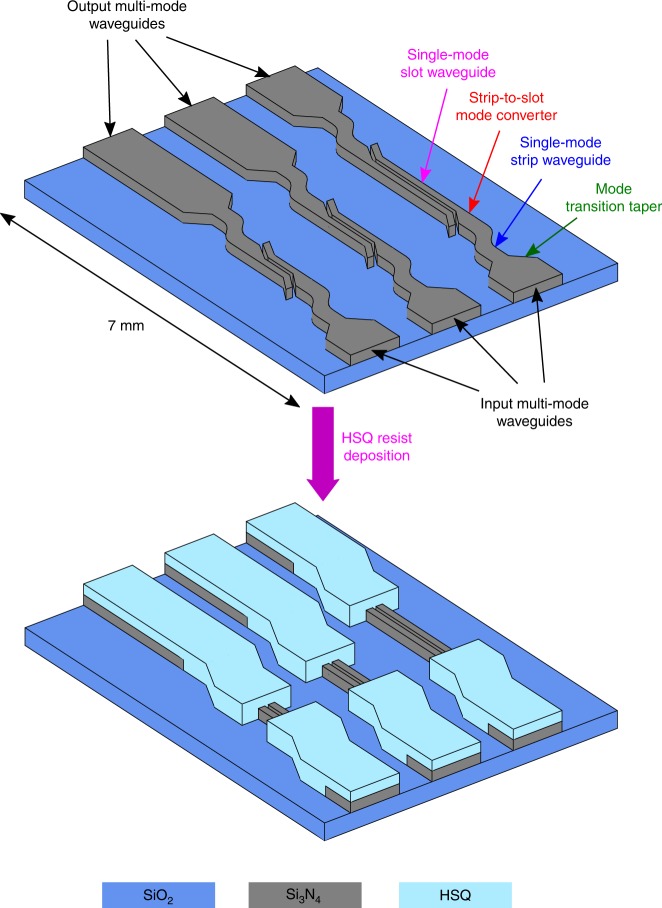
Schematic of the silicon nitride waveguide chip design. The waveguide chip is 7 mm in length and it contains several waveguide channels with different dimensions. Each waveguide channel contains one single-mode slot waveguide, followed by a multi-mode strip waveguide, a multi-mode to single-mode transition taper, a single-mode strip waveguide and a linear strip-to-slot mode converter at the input and output sides of the corresponding channel. The strip waveguides, transition tapers and mode converters were covered with a HSQ-resist to protect them with the succeeding gain layer deposition

Figure 1 presents a schematic of the waveguide chip design. The waveguide chip is 7 mm in length and it contains several waveguide channels with different dimensions (81 in total). An example set of waveguide channels is presented in the schematic. Each waveguide channel contains one single-mode slot waveguide, followed by a multi-mode strip waveguide (4 μm in width), a multi-mode to single-mode transition taper, a single-mode strip waveguide and a linear strip-to-slot mode converter at the input and output sides of the corresponding channel. In this way, the on/off-chip coupling is done into/from the multi-mode strip waveguides to increase the coupling efficiency. Once coupled, the multi-mode to single-mode transition taper converts the multi-mode beam into a single-mode beam. The single-mode beam is then guided by the single-mode strip waveguide into the strip-to-slot mode converter, where the mode is converted suitable for the single-mode slot waveguide that follows. Together with Er-doped Al_2_O_3_, the single-mode slot waveguide provides means for efficient on-chip amplification, as demonstrated in this work. When the optical mode has transmitted through the single-mode slot waveguide, the guiding and transition process is reversed to convert the beam back to its original multi-mode state. Finally, the beam is out-coupled from the output multi-mode strip waveguide.

Prior to the deposition of the Er:Al_2_O_3_ active material, the coupling circuit, i.e., the strip waveguides, the mode transition tapers and the mode converters were covered with a 800 nm thick layer of hydrogen silsesquioxane (HSQ, *n* ~1.45) to prevent their coating with the active material, as shown in Fig. 1. The HSQ cladding was deposited by spin coating and an additional lithography step was performed to form openings for the slot waveguides. The HSQ-covered waveguide chip was then coated with a 150 nm thick layer of Er:Al_2_O_3_ by sequentially depositing Er_2_O_3_ and Al_2_O_3_ onto the waveguide chip with ALD. During the ALD-process, the Er:Al_2_O_3_ layer grows uniformly and conformally on the single-mode slot waveguides as well as on the HSQ-covered coupling circuits. However, the proposed design allows the propagating mode to interact with the gain layer only in the single-mode slot waveguides where the HSQ cladding is absent (as shown by Fig. 1). We chose atomic-layer-deposited Er:Al_2_O_3_ as the gain material because it not only exhibits one of the highest photoluminescence lifetime-density products (see Supplementary Table 1 in Supplementary Note 1), but can also be processed at relatively low substrate temperatures (250–325 °C)^18^. In addition, we have also shown that ALD-oxides (e.g., Al_2_O_3_, TiO_2_, and Al:TiO_2_) are excellent materials in silicon photonic applications due to their exceptional film quality and conformality on various structures, including strip and slot waveguides, couplers and nanobeams^19–27^. For example, ALD–Al_2_O_3_, produced from trimethylaluminium and water, is one of the most popular oxides in photonic, electronic, and optoelectronic applications, and its deposition process has been optimized very carefully throughout the years since its first demonstration in 1989^28,29^. Today, ALD–Al_2_O_3_ is one of the fastest ALD-processes available (up to 1 nm/min) and its deposition temperature can be as low as 20 °C^30^, which has made ALD–Al_2_O_3_ a cornerstone material in CMOS-technology^31–33^.

**Fig. 2:**
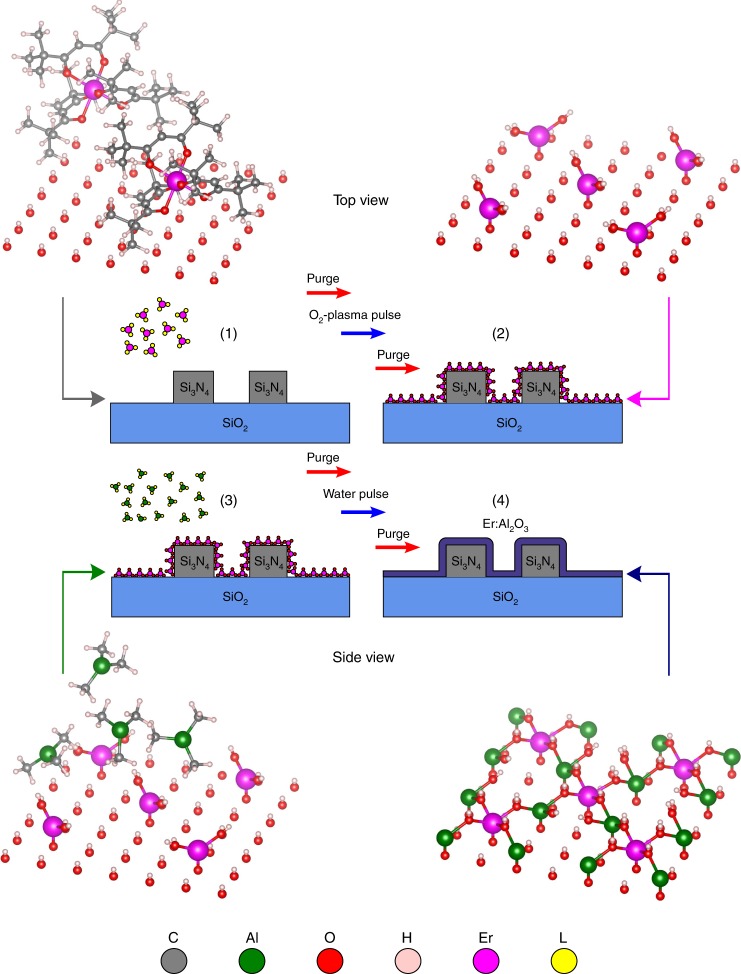
ALD-process sequence for the Er_2_O_3_–Al_2_O_3_ supercycle: (1) Er(thd)_3_ pulsing on the preprocessed Si_3_N_4_ slot waveguide. (2) Resulting device layer after nitrogen purging, oxygen plasma pulsing, and nitrogen purging. (3) TMA-pulsing on the Er_2_O_3_–Si_3_N_4_ surface. (4) Resulting device layer after nitrogen purging, water pulsing, and nitrogen purging. The colors for the individual atoms/molecules are as follows: C: gray; Al: green; O: red; H: white; Er: magenta; thd/methyl ligand (L): yellow

The layer-by-layer sequence of the Er:Al_2_O_3_ gain layer deposition is presented in Fig. 2. The ALD process was initialized by pulsing erbium tris(2,2,6,6-tetramethyl-3,5-heptanedionate) ≡ Er(thd)_3_ onto the hydroxyl-terminated surface of the silicon nitride waveguide (step 1). During the pulsing, the Er(thd)_3_-molecules undergo ligand exchange with the hydroxyl surface sites and hence, adsorb on the waveguide surface. When the ligand exchange occurs, either one or two ligand(s) (thd ≡ C_11_H_19_O_2_) of the Er(thd)_3_ are detached from the precursor but one or two ligand(s) still remain(s) bound to the precursor molecule. Now, as the size of the thd-ligand is very large (>5 Å), it prevents nearby Er(thd)_3_-molecules to adsorb on the surface via the process known as steric hindrance. The steric hindrance forces the nearby Er(thd)_3_-molecules to find surface sites that are separated sufficiently because the ligands can not occupy the same space due to repulsive forces between the overlapping electron clouds. When all the possible surface sites have been filled, step (1) finishes and a purge step follows. In step (2) of the ALD process, the remaining organic ligands are removed or burnt via oxygen plasma. When all the organic ligands have been removed, the oxygen plasma pulse finishes and another purge step follows. The resulting surface is a submonolayer of Er_2_O_3_ where the distance between the individual Er-ions is relatively long and determined by the size of the thd-ligand. This is the core idea of our ALD-deposition: to deposit optically active Er-ions with controlled distance, rather than having a monolayer of Er_2_O_3_ where the majority of the Er-ions are optically inactive due to the very large density of Er-ions in Er_2_O_3_. In step (3) of the ALD-process, trimethylaluminum ≡ TMA is pulsed on the surface and similar reactions occur as in step (1).

Now, as the methyl-ligand (≡CH_3_) of the TMA precursor is much smaller than that of the thd-ligand in Er(thd)_3_, more precursor is bound onto the surface. Majority of the TMA molecules find their way onto the surface sites that could not be filled during step (1), but some may bind onto the anionic sites of the Er_2_O_3_-molecules. When all the possible surface sites have been filled, step (3) ends and a purge step follows. In step (4) of the ALD process, water is pulsed on the surface to undergo ligand exchange with the remaining methyl ligands and thus, stable Al–O bonds are formed. Finally, the last nitrogen purge step ends the Er_2_O_3_–Al_2_O_3_ supercycle. The resulting film is a submonolayer of Al_2_O_3_ where some of the cationic sites have been substituted by the Er-ions. Hence, one supercycle of Er_2_O_3_–Al_2_O_3_ produces a submonolayer of Er-doped Al_2_O_3_. As one cycle of the TMA+ water process is also incapable of forming a monolayer, the Al_2_O_3_-cycle is usually repeated multiple times. In this way, we can further control the Er-distribution to ensure sufficient separation between the Er-ions in the lateral direction. Here, our Er:Al_2_O_3_ is formed by depositing one sub-monolayer of Er_2_O_3_, followed by two sub-monolayers of Al_2_O_3_ and this sequence is repeated 830 times to form a 150 nm thick Er:Al_2_O_3_. The ALD window (i.e., the temperature range for a constant deposition rate) for this process was found to be 250–325 °C. However, we decided to deposit the Er:Al_2_O_3_ film at 300 °C to minimize the amount of impurities in the film while still avoiding the decomposition of the TMA precursor. After the gain layer deposition, the Er:Al_2_O_3_ film was characterized with photoluminescence, Raman and energy-dispersive X-ray spectroscopy (see Supplementary Figs. 1 and 2, as well as Supplementary Table 2 in Supplementary Note 2). These studies show that the Er:Al_2_O_3_ film exhibits amorphous characteristics and the resulting film composition contains ~4.9 × 10^21^ cm^−3^ of Er-ions.

**Fig. 3:**
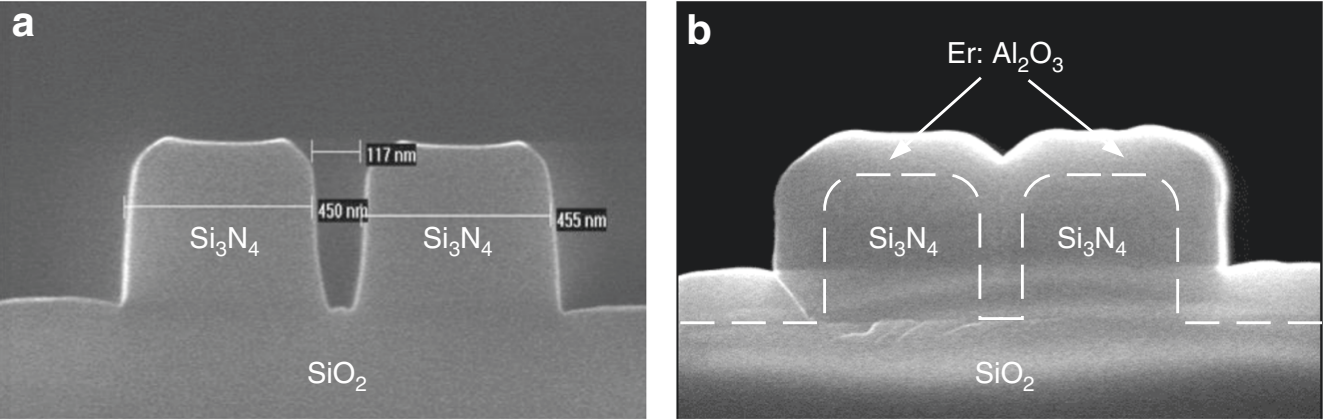
Cross-sections of the fabricated slot waveguides. Silicon nitride slot waveguide with slot width of ~100 nm and strip size of ~460 × 460 nm^2^ (**a**) before and **b** after the Er:Al_2_O_3_ deposition. Cross-sections were measured with scanning electron microscopy

Figure 3a, b shows the cross-sections of the fabricated passive (Si_3_N_4_) and hybrid (Er:Al_2_O_3_–Si_3_N_4_) slot waveguide structures, respectively. In Fig. 3a, the observed slope in the inner walls of the Si_3_N_4_ slot waveguide was attributed to the necessary compromises made in the dry-etching process step parameters, with no penalty to the waveguide propagation loss. Furthermore, Fig. 3b demonstrates that the Er:Al_2_O_3_ gain layer can be conformally deposited into the slot region of the Si_3_N_4_ waveguides, which is an important benefit of ALD during the device fabrication.

### Modal analysis

**Fig. 4:**
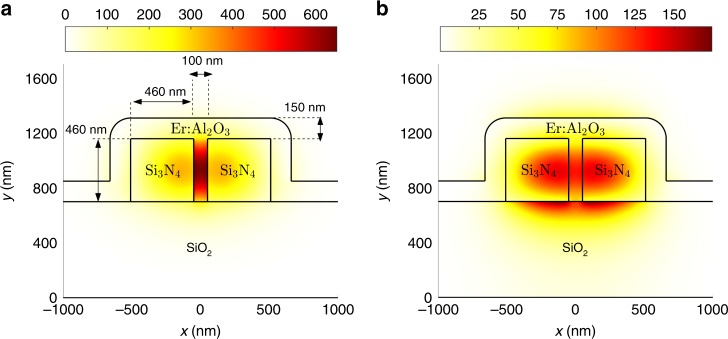
Modal analysis of the hybrid slot waveguides. **a** Simplified cross-section and electric field distribution (|**E**_*x*_|^2^) of the first-order TE-mode at *λ* = 1533 nm of the studied Er:Al_2_O_3_–Si_3_N_4_ hybrid slot waveguides. **b** Electric field distribution (|**E**_*y*_|^2^) of the first-order TM-mode for the corresponding cross-section and wavelength. The following refractive indices were used in the modeling: $n_{{\mathrm{SiO}}_{\mathrm{2}}} = 1.466$, $n_{{\mathrm{Er:Al}}_{\mathrm{2}}{\mathrm{O}}_{\mathrm{3}}} = 1.650$, $n_{{\mathrm{Si}}_{\mathrm{3}}{\mathrm{N}}_{\mathrm{4}}} = 1.997$

From the designed device chip that contains a range of waveguides, we studied the generation of optical gain in three different hybrid slot waveguides with lengths of 250, 700, and 1200 μm. In addition, we studied 1.01, 1.44, and 1.92 cm long slot waveguides before and 0.22, 0.32, and 0.40 cm long slot waveguides after the gain material deposition to accurately determine the propagation losses of the uncoated and hybrid slot waveguides. Figure 4a shows the simplified cross-section for the fabricated hybrid slot waveguides as well as the electric field distribution (|**E**_*x*_|^2^) of the first-order transverse electric (TE) mode at *λ* = 1533 nm. In addition, Fig. 4b shows the electric field distribution (|**E**_*y*_|^2^) of the first-order transverse magnetic (TM) mode at *λ* = 1533 nm for the hybrid slot waveguide cross-section. The electric field distributions of the fundamental modes show that both polarizations can be confined within the waveguide cross-section. However, the TE-mode is more confined in the slot region where the gain medium has been deposited. Thus, in order to produce as high gain as possible, we operate the hybrid slot waveguides by coupling only the TE-polarization into the device. The mode confinement factor of the fundamental TE-mode with the active region of the hybrid slot waveguide was calculated to be *Γ* ≈ 0.311 at *λ* = 1470 nm and *Γ* ≈ 0.315 at *λ* = 1533 nm, respectively (see the analysis with the method in Ref. ^34^ and Supplementary Figs. 3 and 4 in Supplementary Note 3).

### Transmission measurements

**Fig. 5:**
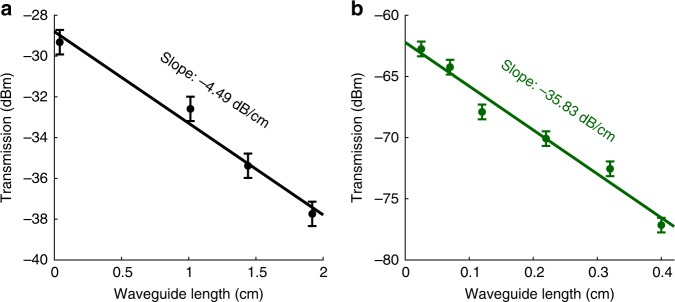
Transmission characterization of the uncoated and hybrid slot waveguides. Transmission of the signal beam (*λ*_c_ = 1533 nm) at the output of **a** 0.04, 1.01, 1.44, and 1.92 cm long uncoated Si_3_N_4_ slot waveguides and **b** 0.025, 0.070, 0.120, 0.220, 0.320, and 0.400 cm long Er:Al_2_O_3_–Si_3_N_4_ hybrid slot waveguides. In **a** and **b**, the solid points correspond to the experimentally measured transmission values with ±0.60 dB signal level accuracy, whereas the solid lines are the theoretical linear least-squares fits to the transmission data with *R*^2^ = 0.9797 and *R*^2^ = 0.9726, respectively

Figure 5a presents the transmission of the signal beam (*λ*_c_ = 1533 nm, *P*_in_ = −13 dBm ≡ 5.0 μW) at the output of four uncoated Si_3_N_4_ slot waveguides of different lengths. With linear least-squares fitting, an average propagation loss of *α*_0,uc_ ≈ 4.49 ± 0.98 dB/cm was calculated for the uncoated waveguides. Figure 5b presents the transmission of the signal beam (*P*_in_ = −45 dBm ≡ 0.03 μW) at the output of six hybrid slot waveguides of different lengths. By applying the same fitting procedure as for the uncoated waveguides, an average propagation loss of *α* ≈ 35.83 ± 4.18 dB/cm was calculated for the hybrid waveguides. The dramatic increase in the propagation loss of the hybrid waveguides can be attributed to the absorption of the large density of Er-ions that remain in their ground state in the absence of pumping. To confirm that the increase in the propagation loss of the hybrid waveguides indeed results from the Er-absorption and not from the Al_2_O_3_ crystal, the background loss the ALD–Al_2_O_3_ was measured from a reference sample (see Supplementary Fig. 5 in Supplementary Note 4). We found $\alpha _{{\mathrm{Al}}_{\mathrm{2}}{\mathrm{O}}_{\mathrm{3}}} = 0.62 \pm 0.01\,{\kern 1pt} {\mathrm{dB/cm}}$ at *λ* = 1533 nm. Thus, Er-absorption was identified as the major loss mechanism in the hybrid waveguides.

### Gain measurements

**Fig. 6:**
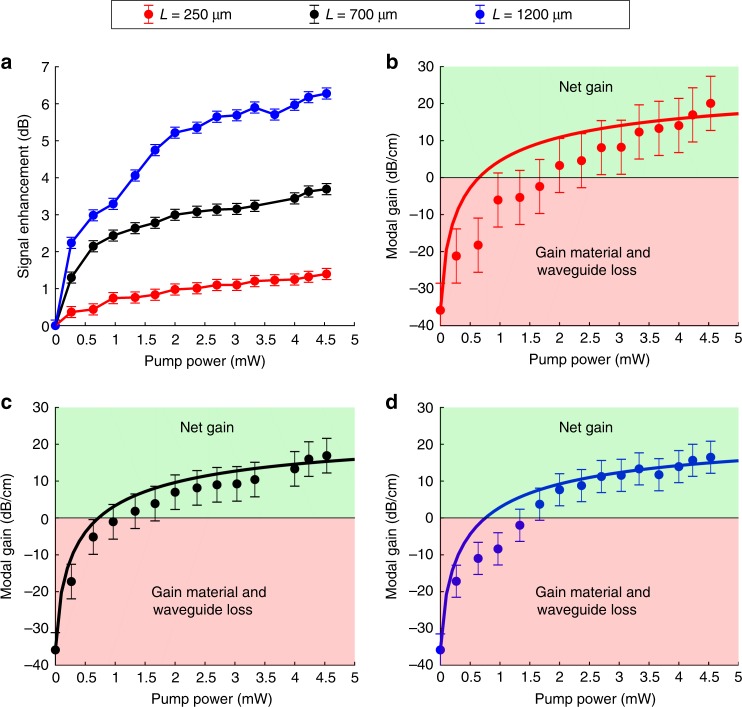
Gain characterization of the hybrid slot waveguides. **a** Measured signal enhancements produced by the 250, 700, and 1200 μm long Er:Al_2_O_3_–Si_3_N_4_ hybrid slot waveguides; Calculated (solid points) and simulated (solid line) modal gain as a function of the injected pump power in **b** the 250 μm long; **c** the 700 μm long, and **d** the 1200 μm long Er:Al_2_O_3_–Si_3_N_4_ hybrid slot waveguides for a −33 dBm (≡0.5 μW) input signal beam. In **a**, the measurement level accuracy is ±0.15 dB. In **b**–**d**, the modal gain of each waveguide (including the error level) has been calculated according to Eqs. (1) and (2) (see Methods), and the regions for the net modal gain and loss are shown in green and red, respectively

Figure 6a presents the measured signal enhancements of the 250, 700, and 1200 μm long hybrid slot waveguides as a function of the injected pump power (*λ*_c_ = 1470 nm) for a 0.5 μW launched signal beam with *λ*_c_ = 1533 nm. During the signal enhancement measurements, we detected the presence of amplified spontaneous emission, which was subtracted from the measured signal level (see the analysis and Supplementary Fig. 6 in Supplementary Note 5). Thus, Fig. 6a shows only the signal enhancement generated by the stimulated emission of the signal beam. As expected, more signal enhancement can be generated as the waveguide length increases. However, higher signal enhancement does not necessarily indicate improved performance from the longer waveguides since the propagation loss also increases with respect to the waveguide length. Therefore, Eq. (1) (see Methods) was used to calculate the net modal gain (in dB/cm) of the hybrid waveguides. The net modal gain generated by each hybrid waveguide is presented in Fig. 6b–d as a function of the injected pump power, respectively. In Fig. 6b–d, the solid points correspond to the experimentally measured values whereas the solid lines are theoretical simulations based on the hybrid waveguide modeling (see the analysis and Supplementary Table 2 in Supplementary Note 6). For all the three hybrid waveguides, we observed that net modal gain was already realized when the pump power in the waveguides reached approximately 1.5–2.0 mW. When the pump power was increased further, the modal gain started to saturate and up to 20.1 ± 7.31 dB/cm net modal gain could be provided with 4.5 mW injected pump power. The highest net modal gain per unit length was measured from the shortest hybrid waveguide with slight decline as the waveguide length increased. The reason for such decline is the requirement for higher pump power in the longer waveguides not only because the gain medium length increases but also as a result of the reduction in the pump efficiency.

To estimate the internal performance of the Er:Al_2_O_3_ gain material in the hybrid slot waveguides, material gain is a more representative characteristic over modal gain since only a fraction of the propagating mode interacts with the gain medium in the hybrid waveguides. Furthermore, the portion of the mode that does not propagate in the active medium experiences an additional passive loss component due to scattering on the Er:Al_2_O_3_–Si_3_N_4_ boundary of the waveguide, which also influences the maximum material gain. To calculate the net material gain of the active layer, the passive propagation loss (≡*α*_0_) of the hybrid waveguides needs to be known (Eq. (3), see Methods). For the uncoated waveguides, *α*_0_ ≡ *α*_0,uc_. However, for the hybrid waveguides, *α*_0_ ≠ *α*_0,uc_ since the value of *α*_0_ is influenced not only by the modal and material properties, but also by the surface roughness of the hybrid waveguides—all of which have changed after the gain material deposition. As a consequence, it is difficult to give an accurate value for *α*_0_. Therefore, we give a lower limit for the material gain generated by the active layer of each hybrid waveguide by assuming *α*_0_ ≈ 0. Finally, we summarize the gain characteristics of the hybrid waveguides in Table 1. Table 1 shows that the highest net material gain per unit length is also generated in the shortest hybrid waveguide. Although the error levels regarding the modal and material gain in this waveguide are quite large, the order of the error is typical for sub-mm long active waveguides, as previously reported by multiple authors (see Supplementary Table 1 in Supplementary Note 1). The origin of the error is the noise level (±0.15 dB) of the optical spectrum analyzer used in our experimental measurement setup. When the waveguide length increases, the error levels are reduced as more signal enhancement is generated and the signal-to-noise ratio is greatly improved.

**Table 1:** Gain characteristics of the 250, 700, and 1200 μm long hybrid slot waveguides

Waveguide length (μm)	Signal enhancement (dB)	Net modal gain (dB/cm)	Net material gain (dB/cm)
250	1.40 ± 0.15	20.1 ± 7.31	≥63.8 ± 23.2
700	3.69 ± 0.15	16.9 ± 4.70	≥53.7 ± 14.9
1200	6.28 ± 0.15	16.5 ± 4.36	≥52.4 ± 13.8

We also attempted to produce net modal gain in the longer waveguides but did not succeed since insufficient amount of pump power was injected into the waveguides. The reason for such limited amount of pump power was the fiber-to-waveguide edge-coupling process, where majority of the pump power was lost. Therefore, by improving the coupling, i.e., with grating couplers, more overall net gain could be expected from the Er:Al_2_O_3_–Si_3_N_4_ hybrid slot waveguides.

## Discussion

The fabricated Er:Al_2_O_3_–Si_3_N_4_ hybrid slot waveguides studied in this work show superior performance in compensating the passive propagation losses of the Si_3_N_4_ slot waveguides as well as providing an internal net gain over the structures. Although the highest overall net modal and material gain (=1.98 ± 0.52 and 6.29 ± 1.66 dB, respectively) provided by our proposed structures are not yet very large due to short waveguide length, the highest net modal and material gain per unit length (=20.1 ± 7.31 and ≥63.8 ± 23.2 dB/cm, respectively) are very considerable. To our knowledge, this is the highest net modal and material gain per unit length measured from erbium-based planar waveguides integrated on silicon (see Supplementary Table 1 in Supplementary Note 1). We believe the reason for such high performance is the well-optimized ALD-process that allows the deposition of high-quality gain layer with controlled erbium-distribution. Furthermore, when it comes to on-chip integration, there are several merits that make the proposed hybrid waveguides very exceptional when compared to traditional erbium-based waveguides. Firstly, the active layer can readily be deposited on passive silicon integrated waveguides with a single process run, avoiding additional on-chip processing^9,10^. Such process can also be straightforwardly adapted to other types of waveguides (e.g., strip waveguides). Secondly, no external couplers (i.e., couplers between the erbium-doped waveguide and the silicon integrated circuit) are needed when the signal light is coupled into and from the active device^35,36^. Finally, unlike most fabrication methods for erbium-based materials, the ALD process can be performed at relatively low temperature (300 °C), enabling CMOS-compatible processing.

On the other hand, the highest net material gain per unit length (i.e., ≥63.8 ± 23.2 dB/cm) measured in this work is more or less in line with those reported from single-crystal erbium silicate (ES) and erbium chloride silicate (ECS) nanowires, which have recently been under intense research due to their very high-erbium solubility (up to 1.62 × 10^22^ cm^−3^) and large peak absorption/emission cross-section (up to 1.24 × 10^−20^ cm^2^). For example, Sun et al. have demonstrated an internal net gain of ~0.69 ± 0.30 dB over a 56 μm long ECS nanowire, which corresponds to 122 ± 53 dB/cm net gain per unit length^37^. Furthermore, for a slightly different compound, Wang et al. have reported an internal net gain of ~0.80 ± 0.32 dB over a 40 μm long silicon–erbium ytterbium silicate nanowire, corresponding to an internal net gain of 200 ± 80 dB/cm^38^. Although both these reports show very promising potential in using the ES/ECS compounds as an active material for silicon integrated photonics, there are several issues that make their integration with silicon integrated circuits very questionable. For example, the ES/ECS compounds, especially in the form of nanowires, require very high growth temperatures (>1000 °C), which completely prevents their integration within a CMOS process. In addition, the crystalline nature of the ES/ECS compounds allows gain to be provided only at the very resonant wavelength (~1532–1533 nm) of the erbium-ions, and the very high-erbium concentration leads to the demand for an enormous amount of pump power to reach population inversion inside the compound. In this work, we can not only produce similar amount of net material gain, but our approach is also fully compatible with silicon integrated waveguides.

## Methods

### Waveguide measurement setup

**Fig. 7:**
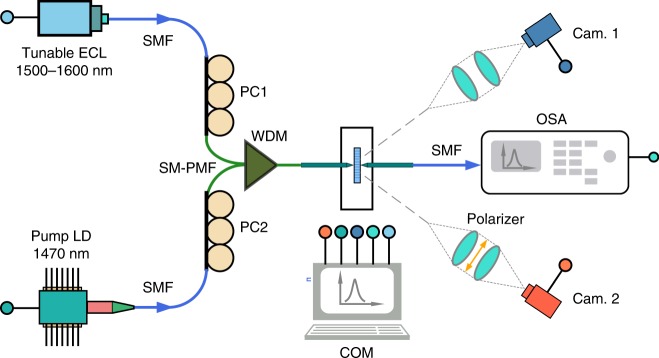
Edge-coupling waveguide measurement setup. The components in the setup are as follows: ECL: external cavity laser, SMF: single-mode fiber, PC: polarization controller, LD: laser diode, WDM: wavelength-division multiplexer, SM-PMF: single-mode polarization-maintaining fiber, Cam: camera, OSA: optical spectrum analyzer, COM: computer

The transmittance and amplification properties of the fabricated slot waveguides were measured with an edge-coupling measurement setup depicted in Fig. 7. Signal and pump light beams were passed through polarization controllers (PC1 and PC2, operation wavelength: 1260–1625 nm and mode field diameter at 1550 nm: 10.4 ± 0.5 μm) and then combined with a 1480/1550 nm wavelength-division multiplexer (WDM, insertion loss at 1470/1533 nm: <1 dB and polarization-dependent loss: ≤0.2 dB) into single-mode polarization-maintaining fiber (SM-PMF, operation wavelength: 1440–1625 nm, maximum insertion loss: 0.5 dB at 1550 nm, minimum extinction ratio at 1550 nm: 23 dB and mode field diameter at 1550 nm: 9.3 ± 0.4 μm). The SM-PMF is stripped and tapered at the output end with a minimum output spot size of 2.5 ± 0.1 μm at a working distance of 14 μm. The signal light was generated from a tunable external cavity laser (*λ*_c_ = 1533 nm, *P*_max_ = 5 mW), whereas an InGaAs laser diode (*λ*_c_ = 1470 nm, *P*_max_ = 144 mW) was used as the pump light source. The combined signal-pump beam was coupled into/from the waveguide chip with the help of two piezo-controlled *xyz*-stages and an optical microscope connected to a camera (Cam. 1). An infrared camera (Cam. 2) and a polarizer were used at the waveguide output before out-coupling was performed to ensure that only the TE-polarization was coupled into the waveguides and to confirm that no stray light was detected at the output (see Supplementary Figs. 7 and 8 in Supplementary Note 5). Finally, the out-coupled spectrum was recorded with an optical spectrum analyzer (OSA, operation wavelength: 600–1750 nm, measurement level: −80 to + 10 dBm at 1250–1700 nm, measurement level accuracy: 0.15 ± 0.02 dB at 1550 nm). The light sources, cameras, and OSA were controlled with a computer (COM).

The fiber-to-waveguide coupling loss of the measurement system was estimated to be ~15 dB (see Supplementary Note 3). In addition, the coupled beam experiences a small loss component (≤0.5 dB) in the input mode converter before transition in the slot waveguide occurs. Thus, a maximum of ~4.5 mW of optical pump power was injected into each slot waveguide. For the signal beam, we measured the output signal change, also known as the signal enhancement (≡SE) as a function of the injected pump power in the slot waveguides to eliminate the coupling losses in the gain evaluation process. The modal gain (*g*_mod_) generated in the slot waveguide was then calculated from the signal enhancement via

1 $$g_{{\mathrm{mod}}}{\mathrm{[dB/cm]}} = {\mathrm{SE}} - \alpha = \frac{{10}}{L}{\mathrm{log}}_{10}\left( {\frac{{S_{{\mathrm{Pump}}\,{\mathrm{on}}}{\mathrm{[W]}} - {\mathrm{ASE[W]}}}}{{S_{{\mathrm{Pump}}\,{\mathrm{off}}}{\mathrm{[W]}}}}} \right) - \alpha ,$$

where *L* is the waveguide length (in cm), *S*_Pump on_ and *S*_Pump off_ are the measured signal power in the presence and absence of pumping, respectively, ASE is the signal power resulting from amplified spontaneous emission and *α* the total propagation loss of the waveguide (in dB/cm) in the absence of pumping. We also calculated the error in the modal gain via

2 $$\delta g_{{\mathrm{mod}}} = \sqrt {(\delta {\mathrm{SE}})^2 + (\delta \alpha )^2} ,$$

where *δ*SE and *δα* are the error levels in the signal enhancement and absorption loss measurements, respectively. In addition, the material gain of the active layer was calculated from the modal gain via

3 $$g_{{\mathrm{mat}}}{\mathrm{[dB/cm]}} = \frac{g_{{\mathrm{mod}}} + \alpha _0}{\Gamma},$$

where *Γ* is the mode confinement factor of the signal beam with the active layer and *α*_0_ is the passive propagation loss (in dB/cm) resulting from the silicon nitride waveguide, i.e., scattering in Si_3_N_4_ and on the Er:Al_2_O_3_–Si_3_N_4_ boundary.

### Code availability

The custom software code used for the theoretical modeling within this manuscript is available from the corresponding author upon reasonable request.

## Supplementary information

Supplementary Information

## Data Availability

The experimental and theoretical data presented within this manuscript is available from the corresponding author upon reasonable request.

## References

[1] Vivien L (2013). Handbook of Silicon Photonics.

[2] Deen MJ, Basu PK (2012). Silicon Photonics: Fundamentals and Devices.

[3] Soref R (2006). The past, present, and future of silicon photonics. IEEE J. Sel. Top. Quantum Electron..

[4] Stampoulidis L (2010). The European BOOM project: silicon photonics for high-capacity optical packet routers. IEEE J. Sel. Top. Quantum Electron..

[5] Fang Z, Chen QY, Zhao CZ (2013). A review of recent progress in lasers on silicon. Opt. Laser Technol..

[6] Bradley JD, Pollnau M (2011). Erbium-doped integrated waveguide amplifiers and lasers. Laser Photonics Rev..

[7] Vázquez-Córdova S (2014). Erbium-doped spiral amplifiers with 20 dB of net gain on silicon. Opt. Express.

[8] Bradley JD (2014). Monolithic erbium- and ytterbium-doped microring lasers on silicon chips. Opt. Express.

[9] Purnawirman J (2013). C- and L-band erbium-doped waveguide lasers with wafer-scale silicon nitride cavities. Opt. Lett..

[10] Hosseini ES (2014). CMOS-compatible 75 mW erbium-doped distributed feedback laser. Opt. Lett..

[11] Bradley JD (2010). Gain bandwidth of 80 nm and 2 dB/cm peak gain in Al_2_O_3_:Er^3^ optical amplifiers on silicon. J. Opt. Soc. Am..

[12] Thomson RR, Psaila ND, Beecher SJ, Kar AK (2010). Ultrafast laser inscription of a high-gain Er-doped bismuthate glass waveguide amplifier. Opt. Express.

[13] Yan YC, Faber AJ, de Waal H, Kik PG, Polman A (1997). Erbium-doped phosphate glass waveguide on silicon with 4.1 dB/cm gain at 1.535 μm. Appl. Phys. Lett..

[14] Kahn A (2008). Amplification in epitaxially grown Er:(Gd,Lu)_2_O_3_ waveguides for active integrated optical devices. J. Opt. Soc. Am. B.

[15] Patel FD, DiCarolis S, Lum P, Venkatesh S, Miller JN (2004). A compact high-performance optical waveguide amplifier. IEEE Photonics Technol. Lett..

[16] Romero-Garcia S, Merget F, Zhong F, Finkelstein H, Witzens J (2013). Silicon nitride CMOS-compatible platform for integrated photonics applications at visible wavelengths. Opt. Express.

[17] Muñoz P (2017). Silicon nitride photonic integration platforms for visible, near-infrared and mid-infrared applications. Sensors.

[18] Rönn J (2016). Atomic layer engineering of Er-ion distribution in highly doped Er:Al_2_O_3_ for photoluminescence enhancement. ACS Photonics.

[19] Gehl M (2012). Effect of atomic layer deposition on the quality factor of silicon nanobeam cavities. J. Opt. Soc. Am. B.

[20] Erdmanis M (2012). ALD-assisted multiorder dispersion engineering of nanophotonic strip waveguides. J. Light. Technol..

[21] Alasaarela T (2013). High-quality crystallinity controlled ALD TiO_2_ for waveguiding applications. Opt. Lett..

[22] Säynätjoki A (2011). Low-loss silicon slot waveguides and couplers fabricated with optical lithography and atomic layer deposition. Opt. Express.

[23] Alasaarela T, Säynätjoki A, Hakkarinen T, Honkanen S (2009). Feature size reduction of silicon slot waveguides by partial filling using atomic layer deposition. Opt. Eng..

[24] Alasaarela T (2011). Reduced propagation loss in silicon strip and slot waveguides coated by atomic layer deposition. Opt. Express.

[25] Säynätjoki A (2009). Angled sidewalls in silicon slot waveguides: conformal filling and mode properties. Opt. Express.

[26] Karvonen L (2013). Enhancement of the third-order optical nonlinearity in ZnO/Al_2_O_3_ nanolaminates fabricated by atomic layer deposition. Appl. Phys. Lett..

[27] Autere A (2015). Slot waveguide ring resonators coated by an atomic layer deposited organic/inorganic nanolaminate. Opt. Express.

[28] Higashi GS, Fleming CG (1989). Sequential surface chemical reaction limited growth of high quality Al_2_O_3_ dielectrics. Appl. Phys. Lett..

[29] Puurunen RL (2005). Surface chemistry of atomic layer deposition: a case study for the trimethylaluminum/water process Surface chemistry of atomic layer deposition: a case study for the trimethylaluminum/water process. J. Appl. Phys..

[30] Profijt HB, Potts SE, van de Sanden MCM, Kessels WMM (2011). Plasma-assisted atomic layer deposition: basics, opportunities, and challenges. J. Vac. Sci. Technol..

[31] Gerritsen E (2005). Evolution of materials technology for stacked-capacitors in 65 nm embedded-DRAM. Solid State Electron..

[32] Van Hove M (2012). CMOS process-compatible high-power low-leakage AlGaN/GaN MISHEMT on silicon. IEEE Electron Device Lett..

[33] Dalapati G (2007). Electrical and interfacial characterization of atomic layer deposited high-k gate dielectrics on GaAs for advanced CMOS devices. IEEE Trans. Electron Devices.

[34] Robinson JT, Preston K, Painter O, Lipson M (2008). First-principle derivation of gain in high-index-contrast waveguides. Opt. Express.

[35] Agazzi L (2017). Monolithic integration of erbium-doped amplifiers with silicon-on-insulator waveguides. Opt. Express.

[36] Mu, J., de Goede, M., Dijkstra, M., García-Blanco, S. M. Monolithic Integration of Al_2_O_3_ and Si_3_N_4_ for double-layer integrated photonic chips. in *Advanced Photonics 2018 (BGPP, IPR, NP, NOMA, Sensors, Networks, SPPCom, SOF)* ITh1I.1 (Optical Society of America, Zurich, Switzerland, 2018).

[37] Sun H (2017). Giant optical gain in a single-crystal erbium chloride silicate nanowire. Nat. Photonics.

[38] Wang XX (2017). Silicon–erbium ytterbium silicate nanowire waveguides with optimized optical gain. Front. Phys..

