# Supplementary material for: Ultra-high on-chip optical gain in erbium-based hybrid slot waveguides
Source: Nat Commun. 2019 Jan 25;10:432. doi: 10.1038/s41467-019-08369-w (PMC6347631; doi:10.1038/s41467-019-08369-w)
Supplement: Supplementary file 1 — Supplementary Information [file 41467_2019_8369_MOESM1_ESM.pdf]

Supplementary Information: Ultra-high on-chip optical gain in erbium-based hybrid slot waveguides

John Rönn,[*,†] Weiwei Zhang,[‡,¶] Anton Autere,[†] Xavier Leroux,[‡] Lasse Pakarinen,[†] Carlos Alonso-Ramos,[‡] Antti Säynätjoki,[†,§] Harri Lipsanen,[†] Laurent Vivien,[‡] Eric Cassan,[*,‡] and Zhipei Sun[*,†,‖]

†*Department of Electronics and Nanoengineering, Aalto University, Tietotie 3, FI-00076 Espoo, Finland*

‡*Centre for Nanoscience and Nanotechnology (C2N), CNRS, Université Paris-Sud, Université Paris-Saclay, UMR 9001, 91405 Orsay Cedex, France*

¶*Optoelectronics Research Centre, University of Southampton, University Road, Southampton, Hampshire SO17 1BJ, United Kingdom*

§*Institute of Photonics, University of Eastern Finland, FI-80101 Joensuu, Finland*

‖*QTF Centre of Excellence, Department of Applied Physics, Aalto University, FI-00076 Espoo, Finland*

E-mail: john.ronn@aalto.fi; eric.cassan@u-psud.fr; zhipei.sun@aalto.fi

Phone: +358 503549262

Supplementary Note 1: Outline of the previous work on erbium-based integrated waveguides

Supplementary Table 1: Properties of the reported erbium-based planar and nanowire waveguides, including the host crystal, fabrication/doping method, fabrication temperature (T), erbium-concentration (N_0), photoluminescence lifetime-density product ($N_0\tau_2 \times 10^{18}$), material gain (g_{mat}) and waveguide length (L).

Planar waveguides							
Group	Crystal	Method	T(°C)	N_0(%)	$N_0\tau_2$ (cm^{-3}s)	g_{mat} (dB/cm)	L
Bradley *et al.*[1]	Al_2O_3	Sput.	650	0.20	1.52	2.0	5.4 cm
Thomson *et al.*[2]	Bismuthate Gl.	Diff.	-	0.63	–	2.3	8.7 cm
Yan *et al.*[3]	Phosphate Gl.	Sput.	-	0.75	3.00	4.1	1.0 cm
Kahn *et al.*[4]	$(Gd, Lu)_2O_3$	PLD	900	0.60	3.78	5.9	7.0 mm
Patel *et al.*[5]	Phosphate Gl.	Diff.	300	8.00	–	13.7	3.0 mm
Vázquez-Córdova *et al.*[6]	$KGd_xLu_y(WO_4)_2$	LPE	925	6.00	–	13.5 ± 5.2	750 µm
**This Work**	**Al_2O_3**	**ALD**	**300**	**4.90**	**10.0**	**$\geq 63.8 \pm 23.2$**	**250 µm**
						**$\geq 53.7 \pm 14.9$**	**700 µm**
						**$\geq 52.4 \pm 13.8$**	**1200 µm**
Nanowire waveguides							
Sun *et al.*[7]	Cl-Silicate	CVD	1053	∼16	8.75	122 ± 53	56 µm
Wang *et al.*[8]	Si-Y-Silicate	CVD	1150	∼5	14.0	200 ± 80	40 µm

Supplementary Note 2: $Er{:}Al_2O_3$ characterization

We characterized the $Er{:}Al_2O_3$ active material by measuring its photoluminescence (PL) response, Raman spectrum and energy-dispersive X-ray spectrum. The measurements were performed from a reference film (thickness ∼150 nm) deposited on a silicon substrate during the waveguide chip processing. The (normalized) PL spectrum of the sample is presented in Supplementary Fig. 1a. The sample exhibits a wide emission spectrum, resembling the typical response found from Er-doped amorphous media. The emission spectrum was used to calculate the absorption spectrum of the sample by applying the McCumber theory.[9] The absorption spectrum is shown as the red solid line in Supplementary Fig. 1a. Supplementary Figure 1a also shows the locations of the pump (1470 nm) and signal (1533 nm) wavelengths

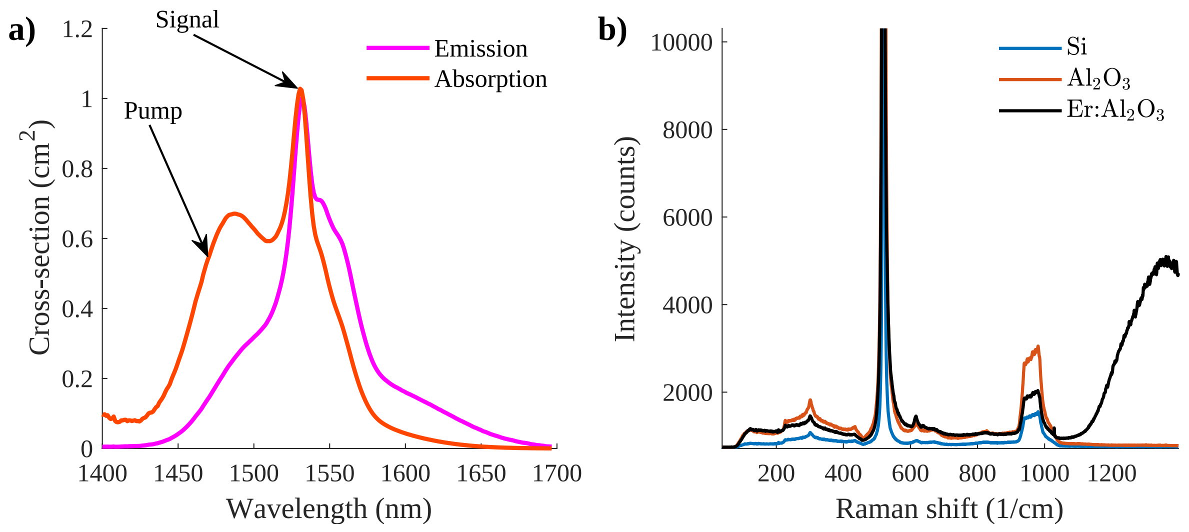

Supplementary Figure 1: a) Measured emission (blue) and calculated absorption (red) cross-section spectra of a 150 nm $Er{:}Al_2O_3$ film (normalized) at $\lambda = 1400 - 1690\,\text{nm}$; b) Measured Raman spectra of a silicon substrate (blue), a 150 nm ALD-Al_2O_3 film on a silicon substrate (red) and a 150 nm $Er{:}Al_2O_3$ film on a silicon substrate (black). The Raman spectra were measured with $\lambda = 488\,\text{nm}$ excitation wavelength.

used in the optical gain characterization of this work.

Supplementary Fig. 1b presents the raman spectrum of the sample. During the Raman measurements, two additional samples were also measured: a reference silicon substrate and a 150 nm Al_2O_3 film deposited on the same silicon substrate. As demonstrated by Supplementary Fig. 1b, all the samples exhibit Raman peaks that are unique to silicon with the exception of a wide peak at around 1360 cm^{-1}. The origin of the peak at 1360 $cm^{-1} \equiv 523\,\text{nm}$ in $Er{:}Al_2O_3$ is the green photluminescence resulting from the $^2H_{11/2} \rightarrow {}^4I_{15/2}$ transition in the Er-ions. Since the raman spectra of the Al_2O_3 film and that of the $Er{:}Al_2O_3$ film are identical aside from the observed green PL, the $Er{:}Al_2O_3$ film is confirmed to share the same (i.e. amorphous) structural properties as the Al_2O_3 film.

The elemental composition of the $Er{:}Al_2O_3$ film was measured with an energy-dispersive X-ray spectrometer (EDX). The EDX spectrum is presented in Supplementary Fig. 2 and the analyzed composition is shown in Supplementary Table 2. The Er-concentration of the $Er{:}Al_2O_3$ film was found to be 4.90 ± 0.52 at. %.

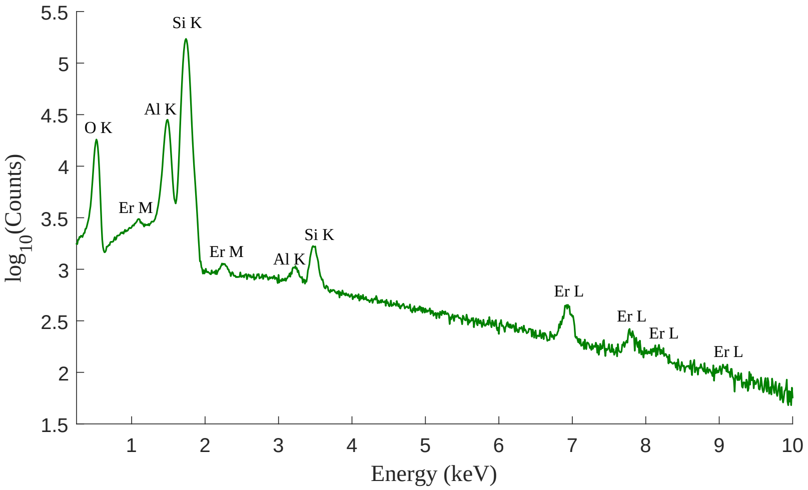

Supplementary Figure 2: EDX-spectrum of the Er:Al_2O_3 material used in this work. The spectrum was measured with an acceleration voltage of 12.5 keV and acquisition time of 100 s.

Supplementary Table 2: Elemental composition of the Er:Al_2O_3 material used in this work. The elemental composition was measured with energy-dispersive X-ray spectroscopy.

Elemental composition (at. %)		
O	Al	Er
63.28 ± 0.63	31.82 ± 0.42	4.90 ± 0.52

Supplementary Note 3: In-depth modal analysis of the hybrid waveguides

We analyze the propagation of the signal and pump beams within the fabricated waveguides by calculating the mode confinement factors with the active region of the waveguides. We also estimate the coupling efficiencies at the corresponding wavelengths.

Mode confinement factor

As derived by Robinson *et al.*[10], the confinement factor for a given mode in the active region (A) of a high-index contrast waveguide is defined as:

$$\Gamma_A = \frac{n_{\text{eff}}^g}{n_A^g} \frac{\iint_A \epsilon \left|\mathbf{E}(x,y)\right|^2 dxdy}{\iint_\infty \epsilon \left|\mathbf{E}(x,y)\right|^2 dxdy}, \tag{1}$$

where n_{eff}^g is the effective group index of the mode, n_A^g the group index of the active region, ϵ the permittivity of the given waveguide material and $\mathbf{E}(x,y)$ is the electric field distribution of the mode for the given cross-section. In this work, the hybrid waveguides are designed to support only the fundamental TE and TM polarizations of the pump and signal beams. Now, as the E_x-component of the fundamental TM-mode is extremely small (see Supplementary Fig. 3), the TM-mode is almost completely eliminated in the coupling process since the hybrid waveguides are operated by coupling only the E_x-component of the signal and pump beams inside the waveguides. Since there are no other modes propagating in the hybrid waveguides, only the TE-mode contributes to the power flow and we can make the approximation $\mathbf{E} \approx \mathbf{E}_x$, where $\mathbf{E}_x$ is the dominant electric-field of the TE-mode. Furthermore, as suggested by Robinson *et al.*, ϵ should be replaced by $d(\omega\epsilon)/d\omega$ in Eq. (1) to account for material dispersion. In this way, we can calculate Γ_A as accurately as possible. By using the

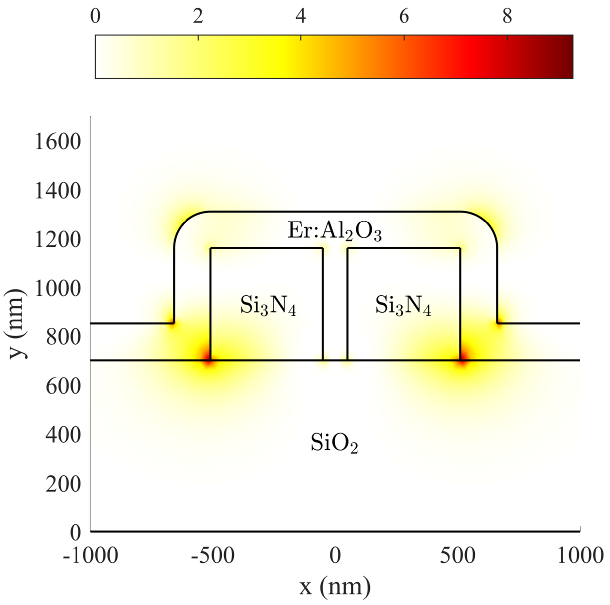

Supplementary Figure 3: $|\mathbf{E}_x|^2$ of the fundamental transverse magnetic (TM) mode for the hybrid waveguide cross-section at $\lambda = 1533\,\mathrm{nm}$.

identity $\epsilon = n^2\epsilon_0$, we express the derivative of ϵ in terms of n as

$$\begin{aligned} \frac{d(\epsilon\omega)}{d\omega} &= \frac{d(n^2\epsilon_0\omega)}{d\omega} = n^2\epsilon_0 + 2n\epsilon_0\omega\frac{dn}{d\omega} \\ &= n\epsilon_0\left(n + 2\omega\frac{dn}{d\omega}\right) = n\epsilon_0\left(2n + 2\omega\frac{dn}{d\omega} - n\right) \\ &= n\epsilon_0\left(2n^g - n\right), \end{aligned} \tag{2}$$

where we have used the definition $n^g = n + \omega dn/d\omega$ for the group index. Finally, we define the mode confinement factor for the TE-mode in our hybrid waveguides as:

$$\Gamma_A = \frac{n^g_{\mathrm{eff}}}{n^g_A}\frac{\iint_A n_A\left(2n^g_A - n_A\right)\left|\mathbf{E}_x(x,y)\right|^2 dxdy}{\iint_\infty n\left(2n^g - n\right)\left|\mathbf{E}_x(x,y)\right|^2 dxdy}, \tag{3}$$

where n and n^g are the refractive and group index profiles of the given waveguide material, respectively and n_A is the refractive index of the active region. To calculate the value of Γ_A, we need to know the refractive indices of Er:Al_2O_3, Si_3N_4 and SiO_2 as a function of

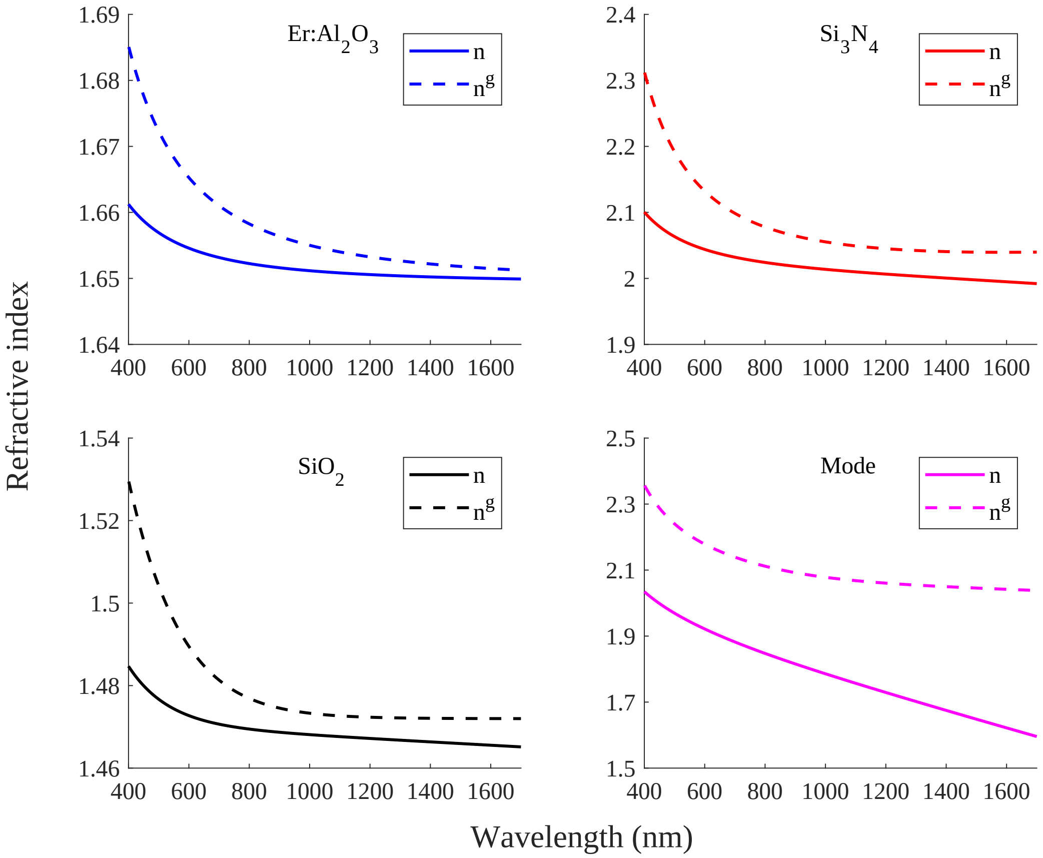

Supplementary Figure 4: Refractive and group index profiles of the waveguide and its materials. Refractive index (solid line) and group index (dashed line) of $Er:Al_2O_3$ (top left); Si_3N_4 (top right) and SiO_2 (bottom left) at $\lambda = 400 - 1700\,\text{nm}$. Effective mode index (solid line) and effective group index (dashed line) of the fundamental TE-mode for the studied waveguide cross-section at $\lambda = 400 - 1700\,\text{nm}$ (bottom right).

the vacuum wavelength. For Si_3N_4 and SiO_2, we use the values tabulated by Gao *et al.* and Luke *et al.*, respectively.[11,12] For $Er:Al_2O_3$, we measured the refractive index profile with spectroscopic ellipsometer at the wavelength range $\lambda = 400 - 1700\,\text{nm}$. The group index of each material was then calculated by using the definition $n_g(\lambda) = n(\lambda) - \lambda dn/d\lambda$. In addition, we determined the effective mode index and the effective group index of the fundamental TE-mode for the corresponding hybrid waveguide cross-section at the same wavelength range. The refractive index profiles are shown in Supplementary Fig. 4. Finally, we calculated the mode confinement factor for the TE-mode to be $\Gamma \approx 0.311$ at $\lambda = 1470\,\text{nm}$ and $\Gamma \approx 0.315$ at $\lambda = 1533\,\text{nm}$, respectively.

Coupling efficiency

Assuming a gaussian spatial profile for the LP_{01}-mode of the signal beam (1533 nm) traveling inside the coupling fiber, we estimate the mode overlap between the signal beam E_1 and the strip waveguide mode E_2 to be

$$\eta = \frac{\left|\iint E_1^*(x,y)E_2(x,y)dxdy\right|^2}{\iint |E_1(x,y)|^2\,dxdy \iint |E_2(x,y)|^2\,dxdy} \approx -6\,\mathrm{dB}. \tag{4}$$

In addition, reflectance at the air-waveguide boundary causes an additional ∼9 dB loss. Thus, the coupling efficiency in our system is approximately –15 dB, which was confirmed experimentally. Similar coupling loss was also estimated at the pump wavelength (1470 nm).

Supplementary Note 4: Al_2O_3 background loss determination

We measured the background loss of our ALD-Al_2O_3 material by depositing a 970 nm thick layer on a transparent 4x4 cm^2 glass substrate at the same process temperature (300 °C) as the Er:Al_2O_3 gain material. A commercial Metricon 2010M prism-coupling system was employed to determine the modal loss of the film. The system excites an evanescent wave that couples into a propagating mode in the lateral direction of the film. A multi-mode fiber then scans the sample in the lateral direction and measures the light scattered from the guided mode. As the light scattered from the surface of the uniform Al_2O_3-film is proportional to the light which remains within the guide, the propagation loss of the guided mode can be calculated. A first-order TE-mode was excited with two laser wavelengths (1064 and 1551 nm) and the scattered intensity was collected as a function of the lateral position of the sample. A linear least-squares fit was applied at both wavelengths and the resulting propagation losses were determined to be 2.43 ± 0.03 and 1.94 ± 0.03 dB/cm at 1064 and

1551 nm, respectively. The measurement results are presented in Supplementary Fig. 5.

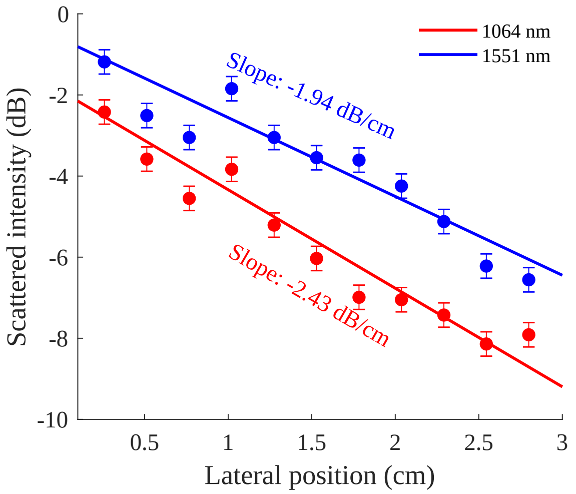

Supplementary Figure 5: Measured scattering intensity (solid points) of the first-order TE-mode in a laterally-guided Al_2O_3-film as a function of the lateral position for 1064 and 1551 nm laser wavelengths and a linear least-squares fit to the data (solid line) with $R^2 = 0.943$ and $R^2 = 0.937$ to determine the propagation loss at 1064 and 1551 nm, respectively.

As the propagation loss caused by the scattering in the Al_2O_3-film is inversely proportional to the fourth power of the wavelength, we can extrapolate the propagation loss at 1533 nm to be $\alpha_{\mathrm{Al_2O_3}} = 1.96 \pm 0.03$ dB/cm. Thus, the contribution of the Al_2O_3 background loss in the hybrid waveguides is $\Gamma_A \alpha_{\mathrm{Al_2O_3}} = 0.62 \pm 0.01\,\mathrm{dB/cm}$.

Supplementary Note 5: Calibration of the waveguide measurement setup

For the sake of providing reliable and accurate measurement results for the gain values of the hybrid waveguides, the waveguide measurement setup needs to be calibrated properly. In general, there are three major components that need to be treated accordingly: amplified spontaneous emission (ASE), stray light and polarization control of the signal and pump beams.

Amplified spontaneous emission

Amplified spontaneous emission is an important phenomenon to consider when one is dealing with high-gain optical amplifiers. When a low-power ($\leq$ 1 µW) signal beam propagates in a strongly-pumped amplifier, the ASE can greatly contribute to the measured signal enhancement. Such measurements with ASE present would give higher values for the signal enhancement and thus, the contribution from the stimulated emission of the signal beam i.e. the actual signal enhancement would be left unknown. The contribution of the ASE can be determined by measuring the signal level at the output of the hybrid waveguides by having only the pump beam present in the waveguides. We demonstrate an example procedure for the 250 µm long hybrid waveguide. When only the pump laser was coupled into the waveguide channel, a wide-bandwidth ASE spectrum was indeed observed at each pump level in the waveguide. Supplementary Figure 6a presents the measured ASE spectrum at the output of the 250 µm long hybrid waveguide channel at the wavelength range $\lambda = 1520 - 1620\,\mathrm{nm}$. The spectra are shown as transmission in the linear scale [W] to highlight their shape more clearly. The shape of the ASE-spectrum differs from that of the typical photoluminescence spectrum of Er:Al_2O_3 as the ASE is affected not only by the hybrid waveguide geometry, but also by the wavelength-dependent amplification and re-absorption by the Er-ions. Similar ASE spectrum has been reported e.g. from Er-Yb-doped optical fibers[13]. To find out how strongly the ASE contributes to the signal enhancement measurements, the output power generated by the ASE was recorded at the signal wavelength ($\lambda = 1533\,\mathrm{nm}$) by averaging the measurement 200 times. The signal laser was then turned on and the signal level was measured at the same wavelength by varying the pump level in the same fashion. The transmitted power levels for these two cases are presented in Supplementary Fig. 6b as a function of the pump power. As can be observed, the ASE can dominate the signal enhancement measurement at high pump levels if the launched power from the signal laser is small (as is the case here). To resolve the actual signal enhancement produced by the stimulated emission of the signal beam, the transmitted power by the ASE was subtracted

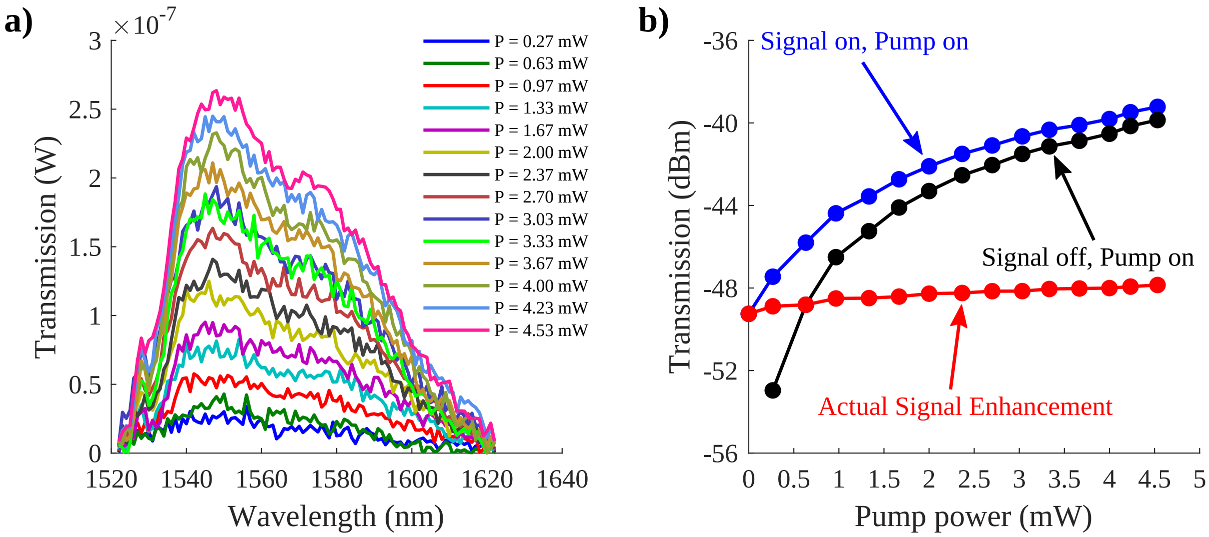

Supplementary Figure 6: a) Measured ASE-spectrum at the output of a 250 µm long hybrid waveguide channel for 13 different pump levels. b) Measured transmission at $\lambda = 1533\,\text{nm}$ at the output of a 250 µm long hybrid waveguide channel as a function of the pump power in two cases: (i) pump laser is on and signal laser is off (black dotted line); (ii) pump and signal laser is on (blue dotted line). The actual signal enhancement produced by the hybrid waveguide (red dotted line) is calculated by subtracting (i) from (ii). Note that all the transmitted signal levels include the ∼15 dB out-coupling loss.

from the total transmitted power. The result is the actual signal enhancement produced by the amplifier, presented as the red dotted line in Supplementary Fig. 6b.

Stray light

If the signal light is not coupled properly into the waveguides during the signal enhancement measurements, some portion of the uncoupled light may travel into the output fiber. This is known as stray light and it can be seen as a dramatic increase in the signal level during the measurements. In general, if the waveguide under study is short in length, the stray light can contribute to the measured signal level due to the fact that the input and output fibers would be separated only by a small distance. Although the hybrid waveguides studied in this work are relatively short in length, each measurement is performed from the relatively long (7 mm) waveguide channels. Thus, the input and output fibers can be separated in our signal enhancement measurements by a large distance, which eliminates majority of the stray

light. In addition, we confirm that no stray light is seen in the infrared camera that is placed at the output of each waveguide channel before the transmission or signal enhancement measurements are performed. An example demonstration is shown in Supplementary Fig. 7. Supplementary Figure 7a shows an infrared image of the signal beam profile after passing

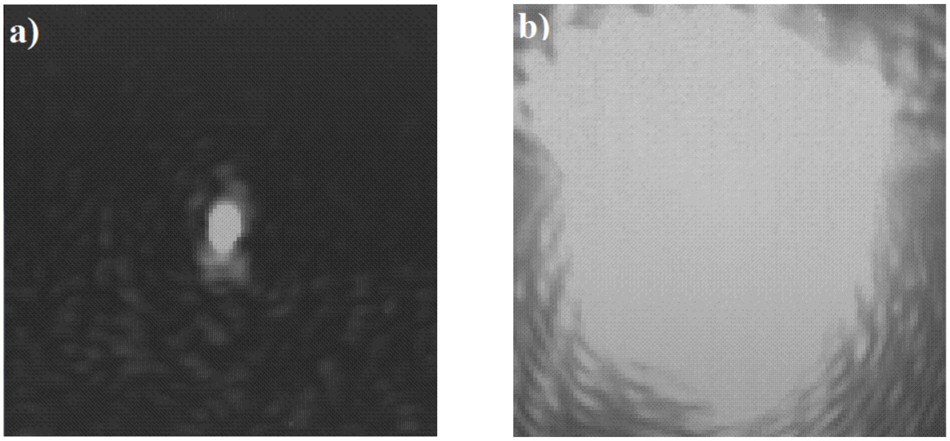

Supplementary Figure 7: Infrared image of the signal beam profile at the output of the 250 µm long hybrid waveguide channel when the input coupling fiber is a) properly aligned and b) misaligned. The beam profiles have been measured after the beam has passed through polarizer that allows only the transmission of the E_x-component of the beam.

through polarizer that allows only the transmission of the E_x-component of the beam. The beam profile has been measured from the 250 µm long hybrid waveguide channel. Figure 7a confirms that no stray light passes into the infrared camera. On the other hand, if the input fiber is tilted slightly upwards, intense stray light can indeed be observed, as demonstrated by Supplementary Fig. 7b. Thus, we invest precise care in the coupling process to eliminate even the slightest misalignment that would lead to unreliable measurement results.

Polarization control of the signal and pump beams

In this work, the hybrid waveguides are operated by coupling only the TE-mode of the pump and signal beams into the waveguides. The polarization of the signal and pump beams needs to be controlled accurately to avoid unwanted polarization state from coupling into the waveguides. In our experimental measurement setup, the fundamental TM-mode

is eliminated via the following procedure. When the pump and signal beams are coupled into the hybrid waveguides during the signal enhancement measurements, the output beams are first guided through a polarizer into an infrared camera. When the polarizer passes

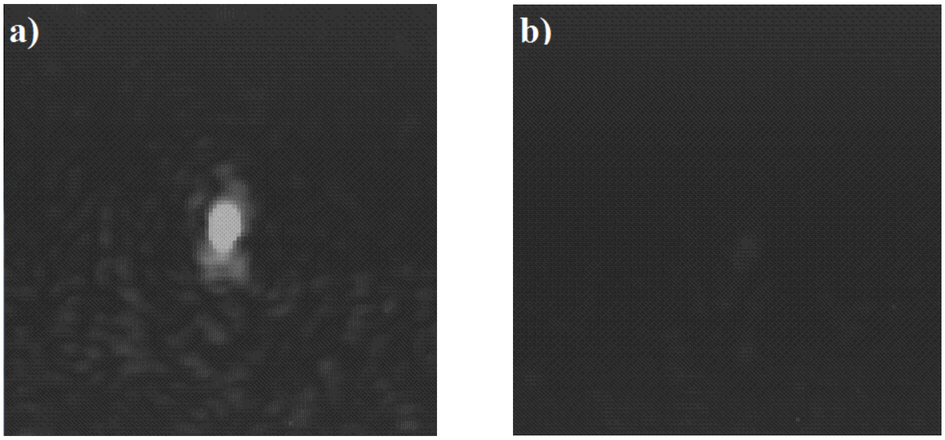

Supplementary Figure 8: Infrared image of the signal beam at the output of the 250 µm long hybrid waveguide channel after passing through polarizer that allows only the transmission of the a) E_x-component of the beam and b) E_y-component of the beam.

only the E_x-component (TE) of each beam (i.e. signal, pump or signal + pump beam), the polarization is controlled so that the highest intensity is seen at the infrared camera. The polarizer is then set to pass only the E_y-component (TM) of the input beams and the polarization of each beam is again controlled so that no light intensity is seen at the infrared camera. This procedure is repeated for the signal, pump and signal + pump beams. The output fiber is then placed close to the waveguide for the actual measurement. An example infrared image of the signal beam profile is presented in Supplementary Fig. 8 for both polarizations after such treatment.

Supplementary Note 6: Hybrid waveguide gain modeling

To model the optical gain in the hybrid waveguides theoretically, we used a 3-level waveguide amplifier model with signal (E_s, P_s, I_s) and pump (E_p, P_p, I_p) beams co-propagating in the

system. The spatially resolved rate equations governing the populations of the active erbium-ions in the first three energy levels of the Er:Al_2O_3 are as follows:

$$\frac{dN_1}{dt} = (R_{21s} + R_{21p} + A_{21})\,N_2 + C_{24}N_2^2 - (R_{12s} + R_{12p})\,N_1, \tag{5}$$

$$\begin{aligned}\frac{dN_2}{dt} &= (R_{12s} + R_{12p})\,N_1 - (R_{21s} + R_{21p} + A_{21} + R_{24})\,N_2 \\ &\quad - 2C_{24}N_2^2 + A_{32}N_3,\end{aligned} \tag{6}$$

$$\frac{dN_3}{dt} = C_{24}N_2^2 + R_{24}N_2 - A_{32}N_3, \tag{7}$$

where $N_i \equiv N_i(x,y,z), A_{ij} \equiv 1/\tau_{ij}$ and R_{ijk} is the transition rate of the signal/pump beam for a given transition $i \to j$, equivalent to

$$R_{ijk} \equiv R_{ijk}(x,y,z) = \frac{\sigma_{ijk}I_{nk}(x,y)P_k(z)}{E_k}, \tag{8}$$

where σ_{ijk} is the absorption/emission cross-section of the Er:Al_2O_3 at the signal/pump wavelength and $I_{nk}(x,y)$ is the normalized transverse intensity distribution of the signal/pump beam. Here, we assume that the excited ions created by the co-operative up-conversion process ${}^4I_{13/2} + {}^4I_{13/2} \to {}^4I_{9/2} + {}^4I_{15/2}$ quickly relax nonradiatively from state ${}^4I_{9/2}$ to lower-lying energy state ${}^4I_{11/2}$. In addition, the co-operative up-conversion ${}^4I_{11/2} + {}^4I_{11/2} \to {}^4I_{15/2} + {}^4F_{7/2}$ and radiative transitions ${}^4I_{11/2} \to {}^4I_{15/2}$ have been neglected due to relatively low population of erbium-ions in state ${}^4I_{11/2}$. We also assume no quenched ions or cross-relaxation present in the system.

Since the total population of the erbium-ions is the sum of the population of the individual states, that is, $N_0 = N_1 + N_2 + N_3$, the rate equations can be solved analytically in the steady-

state regime and N_i can be expressed in terms of N_0:

$$N_1 = \frac{1}{R_{12}} \left(\frac{-b + \sqrt{b^2 - 4ac}}{2a} \right) \left[R_{21} + A_{21} + C_{24} \left(\frac{-b + \sqrt{b^2 - 4ac}}{2a} \right) \right], \tag{9}$$

$$N_2 = \frac{-b + \sqrt{b^2 - 4ac}}{2a}, \tag{10}$$

$$N_3 = \frac{1}{A_{32}} \left(\frac{-b + \sqrt{b^2 - 4ac}}{2a} \right) \left[R_{24} + C_{24} \left(\frac{-b + \sqrt{b^2 - 4ac}}{2a} \right) \right], \tag{11}$$

where

$$a = \frac{R_{12} C_{24}}{A_{32}} + C_{24}, \quad b = \frac{R_{12} R_{24}}{A_{32}} + R_{12} + R_{21} + A_{21}, \quad c = -R_{12} N_0. \tag{12}$$

For a given waveguide cross-section, the transverse intensity distributions of the signal and pump beams are calculated and the spatially resolved rate equations are solved. The spatial evolution of the signal and pump beams in the xyz-plane of the waveguide then follow:

$$\frac{dP_s(x,y,z)}{dz} = P_s(z) I_{ns}(x,y) \left[\sigma_{21s} N_2(x,y,z) - \sigma_{12s} N_1(x,y,z) - \alpha_0 \right], \tag{13}$$

$$\frac{dP_p(x,y,z)}{dz} = P_p(z) I_{np}(x,y) \left[\left(\sigma_{21p} - \sigma_{24} \right) N_2(x,y,z) - \sigma_{12p} N_1(x,y,z) - \alpha_0 \right]. \tag{14}$$

The spectroscopic parameters used in the modeling are given in Supplementary Table 3.

Supplementary Table 3: Spectroscopic parameters used in the gain modeling of the studied hybrid waveguides.

Parameter	Symbol	Value
Er-concentration	N_0	4.9×10^{21} cm^{-3}
Lifetime of state $^4I_{11/2}$	τ_{32}	20 µs
Lifetime of state $^4I_{13/2}$	τ_{21}	2.05 ms
Pump wavelength	λ_p	1470 nm
Signal wavelength	λ_s	1533 nm
Pump abs. cross-section	σ_{12p}	2.875×10^{-21} cm^2
Pump em. cross-section	σ_{21p}	7.495×10^{-22} cm^2
Signal abs. cross-section	σ_{12s}	5.338×10^{-21} cm^2
Signal em. cross-section	σ_{21s}	5.323×10^{-21} cm^2
ETU coeff.	C_{24}	260×10^{-18} s^{-1}cm^{-3}
ESA cross-section	σ_{24}	1×10^{-22} cm^2
Signal power	P_s	0.5 µW
Max. pump power	P_p	4.5 mW

Supplementary References

(1) Bradley, J. D., Agazzi, L., Geskus, D., Ay, F., Wörhoff, K., Pollnau, M. Gain bandwidth of 80 nm and 2 dB/cm peak gain in Al_2O_3:Er^3 optical amplifiers on silicon. *J. Opt. Soc. Am.* **27,** 187–196 (2010).

(2) Thomson, R. R., Psaila, N. D., Beecher, S. J., Kar, A. K. Ultrafast laser inscription of a high-gain Er-doped bismuthate glass waveguide amplifier. *Opt. Express* **18,** 13212–13219 (2010).

(3) Yan, Y. C., Faber, A. J. de Waal, H., Kik, P. G., Polman, A. Erbium-doped phosphate glass waveguide on silicon with 4.1 dB/cm gain at 1.535 μm. *Appl. Phys. Lett.* **71,** 2922–2924 (1997).

(4) Kahn A. *et al.* Amplification in epitaxially grown Er:$(Gd,Lu)_2O_3$ waveguides for active integrated optical devices. *J. Opt. Soc. Am. B* **25,** 1850–1853 (2008).

(5) Patel, F. D., DiCarolis, S., Lum, P., Venkatesh, S., Miller, J. N. A compact high-performance optical waveguide amplifier. *IEEE Photonics Technol. Lett.*, **16,** 2607–2609 (2004).

(6) Vázquez-Córdova, S. A. *et al.* High optical gain in erbium-doped potassium double tungstate channel waveguide amplifiers. *Opt. Express* **26,** 6260–6266 (2018).

(7) Sun, H. *et al.* Giant optical gain in a single-crystal erbium chloride silicate nanowire. *Nat. Photonics* **11,** 589–593 (2017).

(8) Wang, X. X. *et al.* Silicon-erbium ytterbium silicate nanowire waveguides with optimized optical gain. *Front. Phys.* **12,** 1-7 (2017).

(9) McCumber, D.E. Theory of Phonon-Terminated Optical Masers. *Phys. Rev.* **134,** A299-A306 (1964).

(10) Robinson, J. T., Preston, K., Painter, O. and Lipson, M. First-principle derivation of gain in high-index-contrast waveguides. *Opt. Express* **16,** 16659–16669 (2008).

(11) Gao L., Lemarchand F. and Lequime, M. Exploitation of multiple incidences spectrometric measurements for thin film reverse engineering. *Opt. Express* **20,** 15734–15751 (2012).

(12) Luke, K., Okawachi, Y., Lamont, M.R.E., Gaeta, A.R. and Lipson, M. Broadband mid-infrared frequency comb generation in a Si_3N_4 microresonator. *Opt. Lett.* **40,** 4823-4826 (2015).

(13) Williams, C.R.S., Salevan, J.C., Li, X., Roy, R. and Murphy, T.E. Fast physical random number generator using amplified spontaneous emission. *Opt. Express* **18,** 23584-23597 (2010).
